# Supplementary material for: Adaptation of Cyanobacteria to the Endolithic Light Spectrum in Hyper-Arid Deserts
Source: Microorganisms. 2022 Jun 11;10(6):1198. doi: 10.3390/microorganisms10061198 (PMC9228357; doi:10.3390/microorganisms10061198)
Supplement: Supplementary file 1 [file microorganisms-10-01198-s001.zip › microorganisms-1763714-supplementary.pdf]

SUPPLEMENTARY MATERIAL

Adaptation of cyanobacteria to the endolithic light spectrum in hyper-arid deserts

Bayleigh Murray, Emine Ertekin, Micah Dailey, Nathan T. Soulier, Gaozhong Shen, Donald A. Bryant, Cesar Perez-Fernandez, Jocelyne DiRuggiero

Table S1. Metadata and metagenome accession numbers for endolithic isolates

Strain name	Origin	Substrate	Taxonomy	IMG ID
S-NGV-2P1	Timna Park, Negev Desert, Israel	sandstone	*Chroococcidiopsis*	3300039401
G-MTQ-3P2	Monturaqui, Atacama Desert, Chile	gypsum	*Chroococcidiopsis*	3300037877
C-VL-3P3	Valle de la Luna, Atacama Desert, Chile	calcite	*Chroococcidiopsis*	3300039404

Table S2. Gene distribution in the FaRLiP cluster of *Chroococcidiopsis* G-MTQ-3P2

genome locus	function	gene symbol	annotation	protein size (aa)	gene size (bp)
Ga0395813_0004_87482_87718	photosystem II PsbH protein	*psb* H2	KO:K02709	78	234
Ga0395813_0004_87766_89256	photosystem II CP47 chlorophyll apoprotein	*psb* B2	KO:K02704	496	1488
Ga0395813_0004_89765_91165	photosystem II CP43 chlorophyll apoprotein	*psb* C2	KO:K02705	466	1398
Ga0395813_0004_91234_92292	photosystem II P680 reaction center D2 protein	*psb* D3	KO:K02706	352	1056
Ga0395813_0004_92384_92905	FRL-allophycocyanin	*apc* D3	KO:K02095	173	519
Ga0395813_0004_92871_95210	phycobilisome core-membrane linker protein	*apc* E2	KO:K02096	779	2337
Ga0395813_0004_95279_95758	FRL-allophycocyanin	*apc* D2	KO:K02095	159	477
Ga0395813_0004_95873_96358	allophycocyanin beta subunit	*apc* B2	KO:K02093	161	483
Ga0395813_0004_96460_96936	FRL-allophycocyanin	*apc* A2	KO:K02095	158	474
Ga0395813_0004_97067_98065	photosystem II P680 reaction center D1 protein	*psb* A3	KO:K02703	332	996
Ga0395813_0004_98412_99542	photosystem II P680 reaction center D1 protein	*chl* F	KO:K02703	376	1128
Ga0395813_0004_99771_100004	hypothetical protein	hypothetical			
Ga0395813_0004_100391_102259	DNA-binding response OmpR family regulator	*rfp* B	COG0745	622	1866
Ga0395813_0004_102532_105279	light-regulated signal transduction histidine kinase	*rfp* A	COG4251	915	2745
Ga0395813_0004_105417_105794	CheY-like chemotaxis protein	*rfp* C	COG0784	125	375
Ga0395813_0004_106087_106524	CheY-like chemotaxis protein	*che* Y-like	COG0784	145	435
Ga0395813_0004_106961_109309	photosystem I P700 chlorophyll a apoprotein A1	*psa* A2	KO:K02689	782	2346
Ga0395813_0004_109455_111677	photosystem I P700 chlorophyll a apoprotein A2	*psa* B2	KO:K02690	740	2220
Ga0395813_0004_112052_112603	photosystem I subunit 11	*psa* L2	KO:K02699	183	549
Ga0395813_0004_112614_112838	photosystem I subunit 8	*psa* I2	KO:K02696	74	222

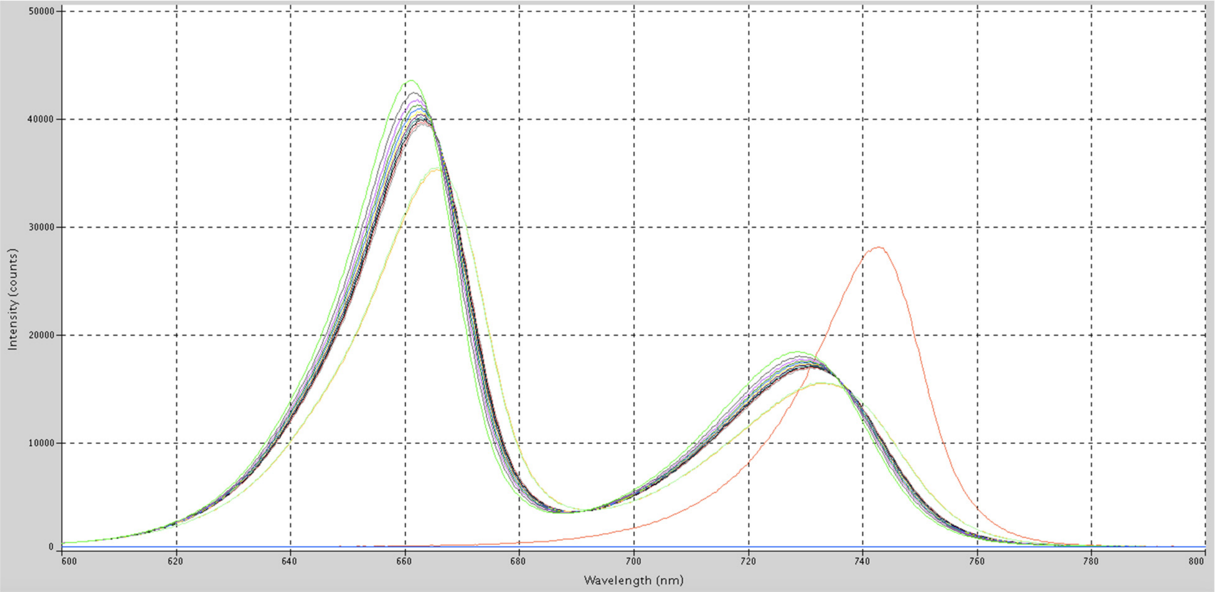

Figure S1. Spectra of white and far-red lights used in growth experiments. The VL spectrum is represented by multiple colored lines with absorption maxima at 665 and 730 nm. The FRL spectrum is represented by the red line with absorption maximum at 745 nm.

```
9212 Chl f synthase          1    [21]TANKLsKRR---KKVNYWEKFCSWVTSTENRLYVGWFGVLMIPCVLTAATV-FIIAIIAAPPVDMDGIGVPISGSI  93
Ga0395813_0004_98412_99542   1    [22]VTNEL-KKR---ESASIWDRFCNWVTSTENRLYIGWFGVLMIPCMLTAASV-FIIAMIAAPAVDMDGMSSPITGSL  93
Ga0395813_0004_97067_98065   1        ----------------------------------------MIP-LLGVSICvFTIAFIAAPPVDIDGIREPVAGSL  35
Ga0395813_0013_94225_95307   1        MTTTL-QRE---RSSSLWDRFCNWITSTENRIYVGWFGVLMIPTLLSATIC-FIIAFIAAPPVDIDGIREPVAGSL  71
Ga0395813_0035_54237_55319   1        MNTIV-QRRpelEIAKVWNRFCEWVTSTDNRIYIGWFGVLMIPTLLTASIC-FILAFAIAPSVDLDGIREPVIGSL  74
Ga0395813_0043_12497_13573   1        MTTTL-QRR---SSANVWDRFCDWIVSIENRLYIGWFGLLMIPTLLAATTC-FIIAFIAAPPVDIDGIREPVAGSL  71
Ga0395813_0043_46650_47348   1        MTTTL-QRR---ESASLWEQFCNWVASTENRLYIGWFGVLMIPTLLAATTC-FIVAFIAAPPVDIDGIREPVAGSL  71

9212 Chl f synthase          94   LSGNNIITAAVVPTSAAIGLHFYPIWEAASIDEWLYNGGPYQLIVLHFLIGIIAYQDREWELSYRLGMRPWISLAFTAPV  173
Ga0395813_0004_98412_99542   94   LDGNNIITAAVVPTSAAIGLHFYPIWEAASLDEWLYNGGPYQLIVLHFLIGIICYQDREWELSYRLGMRPWISLAFTAPV  173
Ga0395813_0004_97067_98065   36   LYGNNIITGAVVPMSNAIGLHFYPIWEAASMDEWLYNGGPYQMIGFHYIPALACYMGREWELSYRLGMRPWIAVAYSAPF  115
Ga0395813_0013_94225_95307   72   IYGNNIISGAVVPSSNAIGLHFYPIWEAASLDEWLYNGGPYQLVIFHFLIGCFCYMGRQWELSYRLGMRPWICVAYSAPL  151
Ga0395813_0035_54237_55319   75   MGGNNLITAAVVPTSAAIGLHFYPLWEAASMDEWLYNGGPYQLIVLHFLIGIWCYLGRLWEVSYRLGMRPWTAVAFSAPA  154
Ga0395813_0043_12497_13573   72   LYGNNIISGAVVPSSNAIGLHFYPIWEAASLDEWLYNGGPYQLVIFHFLIGVFCYLGREWELSYRLGMRPWIAVAYSAPV  151
Ga0395813_0043_46650_47348   72   IYGNNIISGAVVPSSNAIGLHFYPIWEAASLDEWLYNGGPYQLVIFHFLIGVFCYMGREWELSYRLGMRPWICVAYSAPV  151

9212 Chl f synthase          174  AASVSVLLIYPVGQGSLSAGMPLGISGTFHFMLQFQADHNILMSPLHQLGVIGVLGGAFAAAMHGSLVTSTLIRSHN[2]  252
Ga0395813_0004_98412_99542   174  AASISVFLVYPVGQGSFSAGMPLGISGTFNFMLRFQADHNILMSPFHVLGVIGVLGGAFLSAMHGSLVTSTLIRAAN[5]  255
Ga0395813_0004_97067_98065   116  AATSSVFLIYPIGQGSFSDGLPMGISGTFNFMFVFQAEHNILMHPFHMLGVAGVLGGSLFCAMHGSLVTSSLIRETS     192
Ga0395813_0013_94225_95307   152  ASATAVFLIYPIGQGSFSDGMPLGISGTFNFMLVFQAEHNILMHPFHQLGVAAVFGGALFCAMHGSLVTSSLVRETT     228
Ga0395813_0035_54237_55319   155  AAATAVLLVYPIGQGSFADGLPLGIAGTFNFMLAVQADHNILMHPFHMLGVAGVFGGALLSALHGSLVTSTLIRQTQ[1]  232
Ga0395813_0043_12497_13573   152  AAATAVFLIYPIGQGSFSDGMPLGISGTFNFMLVFQAEHNILMHPFHQLGVAGVFGGALFSTMHGSLVTSSLVRETT     228
Ga0395813_0043_46650_47348   152  AAATAVFLIYPIGQGSFSDGMPLGISGTFNFMLVFQAEHNILMHPFHQLGVAGVFGGALFSAMHGSLVTSSLVRETT     228

9212 Chl f synthase          253  ESESINKGYKLGQQHPTYNFRSAQ-VYLWHLIWQRVSF   PNSRKLHFFLAALPVAGIWSAALGVDIAAFDFDYLQFHQ  328
Ga0395813_0004_98412_99542   256  PTESTNTGYKLGQKRPTYSFRAAQ-LYLWRLIWRGTSF   PNSRRLHFFLAAFPVAGIWSAALGVDIAAFNFEKLNFEP  331
Ga0395813_0004_97067_98065   193  DSESQNYGYKFGQESETYNIVAAH-GYFGRLIFQYASF   NNSRSLHFFLAAWPVVCIWAVALGISTMAFNLNGFNFNN  268
Ga0395813_0013_94225_95307   229  ETESQNYGYKFGQEEETYNIVAAH-GYFGRLIFQYASF   NNSRSLHFFLGAWPVVGIWFTALGISTMAFNLNGFNFNQ  304
Ga0395813_0035_54237_55319   233  -HESVNAGYKLGQSQMTYHYLAGHyGFLGRLLVPWFAS   QNHRAFHFFLAALPTIGIWFATAGICSVAFGLNGFNFNH  308
Ga0395813_0043_12497_13573   229  EIESLNNGYKFGQEEETYNIVAAH-GYFGRLVGRTNEI[5]ANSRSLHFLLAIWPVMGIWFTSLGISTMAFNLNGFNFNQ  309
Ga0395813_0043_46650_47348   229  ETESQ---------------------------------   ---------------------------------------  233

9212 Chl f synthase          329  PELKSQGQIIHTWADTIDWASLGIKVLDERHIYDFPENLTAGEVVPWK        376
Ga0395813_0004_98412_99542   332  THIESQGRTVNTWANAIDWANLGIDMARDRQLHQFPTDL---MTVSSE        376
Ga0395813_0004_97067_98065   269  SVLDSQGHVLPTWADVLNRANLGFEVMHERNAHNFPLDLACGDAVPVA[16]    332
Ga0395813_0013_94225_95307   305  SVLDSQGRVVNTWADVLNRANLGMEVMHERNAHNFPLDLASGEATPVA[ 8]    360
Ga0395813_0035_54237_55319   309  SILDSSGRVVRTEADLLNRADLGIQAMHAVNTHHFPNIIAGGGIQPVN[ 4]    360
Ga0395813_0043_12497_13573   310  SILDSQGRVVNTWADILNRANLGMEVMHERNAHNFPLDLASVEA----[ 5]    358
Ga0395813_0043_46650_47348   ------------------------------------------------
```

Figure S2. Protein alignment of Chl *f* synthase from *Chlorogloeopsis fritschii* PCC 9212 (9212 Chl *f* synthase) with Chl *f* synthase identified in the G-MTQ-3P2 (Ga0395813_0004_98412_99542), as well as all annotated *psbA* genes. Ligands to the $Mn_4Ca_1O_5$ cluster are highlighted in light blue. Tyrosine Yz residues are highlighted in yellow. Histidine residues thought to be involved in binding P680 Chl *a* are highlighted in green. Residues thought to ligate an additional Chl *a* are in green font. Residues involved in proton-coupled electron transport are highlighted in pink. Black residues are conserved across all sequences and red residues are not. Ga0395813_0004_98412_99542 lacks any ligands to the $Mn_4Ca_1O_5$ cluster, characteristic of Chl *f* synthase [17].
